# Serum Concentrations of Fipronil and Metabolites in Japanese Pregnant Women: Relationship with Thyroid Hormone Levels

**DOI:** 10.3390/toxics13030213

**Published:** 2025-03-14

**Authors:** Kunishige Ikeda, Aya Hisada, Takamitsu Otake, Ryo Omagari, Daisuke Nakajima, Nobumasa Kato, Jun Yoshinaga

**Affiliations:** 1 Faculty of Life Sciences, Toyo University, 48-1 Oka, Asaka, Saitama 351-8510, Japan; k-ikeda@l.wdb-eu.com; 2 Center for Preventive Medical Sciences, Chiba University, 1-33 Yayoi cho, Inage, Chiba 263-8522, Japan; a_hisada@chiba-u.jp; 3 National Institute of Advanced Industrial Science and Technology, 1-1-1 Umezono, Tsukuba, Ibaraki 305-8563, Japan; t-ootake@aist.go.jp; 4 National Institute for Environmental Studies, 16-2 Onogawa, Tsukuba, Ibaraki 305-8506, Japan; dnakaji@nies.go.jp (D.N.); 41317444@hama-med.ac.jp (R.O.); 5 Neuropsychiatric Research Institute, 91 Bentencho, Shinjuku, Tokyo 162-0851, Japan; katon@med.showa-u.ac.jp

**Keywords:** insecticide, fipronil sulfone, fipronil sulfide, biomarker, thyroxine, thyroid stimulating hormone (TSH), birth cohort, early gestation, food habit

## Abstract

Fipronil, a widely used phenylpyrazole insecticide, is known to disrupt circulating thyroid hormone (TH) levels in rodents. Concentrations of fipronil and its metabolites (fipronil sulfone and fipronil sulfide) in serum samples collected in 2009–2011 were measured for 131 Japanese pregnant women by a sensitive and accurate liquid chromatography-tandem mass spectrometric method developed in our laboratory to relate TH levels. Fipronil sulfone was detected in all the subjects with the median being 21 ng/L (min–max: 6.8–89), but fipronil and fipronil sulfide were detected in none of the subjects (detection limit: 5.0 and 1.2 ng/L, respectively), indicating a rapid and exclusive oxidative conversion to fipronil sulfone upon exposure. The median concentration of fipronil sulfone was lower than those previously reported for general populations in other countries by one order of magnitude. There were no attributes or dietary habits of the subjects that significantly vary the serum fipronil sulfone concentrations. Multiple regression analyses found no significant association between serum concentrations of fipronil sulfone and free thyroxine- or thyroid-stimulating hormone levels, indicating the absence of adverse effects on circulating TH levels probably due to the lower exposure levels of the present subjects. The present result would be valuable for establishing a dose–effect relationship of fipronils in humans on population levels.

## 1. Introduction

Fipronil is one of the phenylpyrazole family insecticides used widely in agriculture, veterinary drugs for pets and livestock, and household pest control from the late 1990’s [1]. Because of its broad spectrum of application, it is detected not only as residue in vegetables and crops for consumption [2], but also in residential environments [3,4,5,6]. Therefore, human exposure to this insecticide is estimated to be common. This is supported by the frequent detection of a major metabolite of fipronil (fipronil sulfone) in serum samples from general populations [7,8,9].

Although fipronil was developed to exert its toxicity selectively on pests, toxicities to non-target organisms including mammals have become apparent. Among several different toxicities of fipronil observed in mammals, the effect on circulating thyroid hormone (TH) levels has been of interest. Leghait et al. [10] observed decreased plasma thyroxine (T4) levels in fipronil-administered rats, which was accompanied by increased thyroid-stimulating hormone (TSH) levels as a result of negative feedback. This decrease in plasma T4 was attributed to elevated T4 metabolism in administered animals. A similar result was reported in Moser et al. [11] A metabolite of fipronil, fipronil sulfone, is also known to have a similar TH-disruption effect. [12] Since TH is essential for the development of tissues and organs of fetuses, the TH disruption in pregnant mothers is of considerable concern for the development of the offspring: even a mild TH deficiency in pregnant mothers resulted in significantly lowered cognitive development of the infants [13]. Thus, the level of fipronil and metabolite (designated as “fipronils” hereafter) exposure in pregnant mothers attracts attention in terms of maternal TH disruption. Moreover, taking into consideration the known transplacental transfer of maternal fipronils [14], maternal exposure to fipronils during pregnancy could also affect the normal development of offspring via the direct disruption of fetal TH by transferred fipronils.

In the previous studies that related fipronil exposure to TH levels in humans, serum or plasma fipronils concentrations have been used as a biomarker of exposure because fipronils were hardly detectable in the urine of humans [7]. Kim et al. [8] found a significant negative association between the levels of fipronil sulfone and those of T3 and free T3 in the sera of umbilical cord blood of Korean newborn babies, indicating an adverse effect of maternal fipronils exposure.

It is the aim of this study to determine serum concentrations of fipronil and its metabolites in Japanese pregnant women by using a sensitive analytical method and to examine the relationship between the levels of TH and fipronils exposure.

## 2. Materials and Methods

The study population of this study was pregnant women of early gestation (10–12 weeks) who attended the Obstetric Department of Showa University Hospital in Tokyo in 2009–2011. The women were enrolled in our birth cohort study on the maternal endocrine environment and offspring development after giving informed consent. The criteria for eligibility were that the woman was of reproductive age (20–50), lived in the Tokyo Metropolitan Area, and had no known diseases that could affect normal thyroid function. The original cohort study was approved by the ethical committee of Showa University, and the present study including the additional analysis of serum for fipronils was approved by the ethical committee of Toyo University (TU2019-K-043).

Blood and urine samples were collected from 231 participants for the assessment of TH status and environmental chemical exposure, and they were stored in a freezer (−20 °C) until analyzed. Of the serum samples collected, the amount of 131 samples was adequate for chemical exposure assessment, and they were analyzed for fipronil and metabolites in this study. The method of determination of fipronil, fipronil sulfone, and fipronil sulfide in the serum by liquid chromatography-tandem mass spectrometry (LC-MS/MS) was developed in our laboratory. To 200 μL of serum, added was 1 mL of acetonitrile (Pesticide Grade, FUJIFILM Wako Pure Chemical Co., Tokyo, Japan) containing 100 pg each of ^13^C-labelled fipronil, fipronil sulfone, and fipronil sulfide, which were prepared from 100 μg/mL stock methanol solutions (Cambridge Isotope Laboratories Inc., Tewksbury, MA, USA), and vortexed for 1 min. Denatured serum protein was removed by centrifugation. The supernatant was loaded on a solid phase extraction cartridge (HybridSPE Phospholipid Cartridge 1 cc/30 mg, Sigma-Aldrich, St. Luis, MO, USA) to remove phospholipids from the serum samples. Acetonitrile in the elute was removed by gentle flow of nitrogen gas, and the elute was finally made up to exactly 200 μL by the addition of purified water.

The LC-MS/MS used was ExionLC AC coupled with Triple Quad5500+ QTRAP Ready (AB Sciex, Tokyo, Japan) operated as presented in Table 1. In this Table, also included, is the optimized condition of LC. The mixed calibration standard (0, 1,5, 10, 50, 100, 200 ng/L) was prepared from a standard stock solution prepared from commercial standards (FUJIFILM Wako Pure Chemical Co., Tokyo, Japan) dissolved in methanol and used with 500 ng/L of mixed ^13^C-labelled surrogates. The concentrations of fipronil, fipronil sulfone, and fipronil sulfide were determined by internal standardization. A commercial serum sample for quality control of biochemical analysis (Control Serum I, lot # 1924_1, FUJIFILM Wako Pure Chemical Co., Tokyo, Japan) was used for our method development, which was found to contain 36 ng/L of fipronil sulfone and undetectable amounts of fipronil and fipronil sulfide by our analysis (mentioned later).

The 131 serum samples from the participants were prepared and analyzed as described above in 9 batches: each batch included one procedural blank, one Control Serum I sample for internal quality control, and 12–15 serum samples from the participants.

Data of serum concentrations of TSH and fT4 used in this study were those determined by electrochemiluminescence immunoassay (ECLIA) with a commercial kit (ECLusys TSH, fT4, Roche, Tokyo, Japan), and those of urinary iodine concentrations were determined by inductively coupled plasma mass spectrometry [15].

Age, pre-pregnancy body mass index (BMI), urinary concentration of iodine (creatinine corrected), parity, smoking by the time of diagnosis of pregnancy, and passive smoking status of the participants were obtained in our cohort study and used as covariates in the multiple regression analysis for the association between fipronils exposure and levels of free T4 and TSH in this study. Of the covariates used in the regression analysis, parity and smoking/passive smoking status were categorical variables, and others were continuous variables. A possible association was examined between serum levels of fipronils and dietary habits of the participants based on a questionnaire asking about the frequency of consumption (none, once a month, once a week, 2–3 times a week, every day, every meal) of major food categories including rice, bread, seafood, seaweed, meat, eggs, and vegetables and fruits. Statistical analyses, including descriptive statistics, a Pearson correlation analysis, an analysis of variance, and a linear multiple regression analysis, were carried out using an SPSS package (ver. 27, IBM, Tokyo, Japan) with log-transformed fipronil sulfone concentrations. *p* = 0.05 was taken as significant throughout the analyses.

## 3. Results and Discussion

### 3.1. Analytical Performance

**Figure 1:**
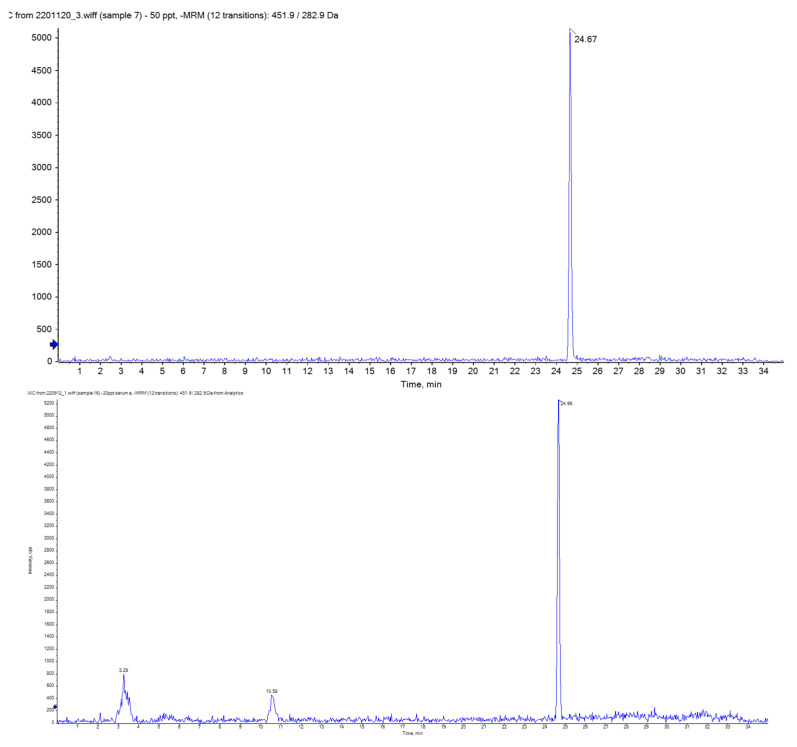
A chromatogram (*m*/*z*: 451 > 282) of the standard solution (fipronil sulfone 50 ng/L, **upper**) and a serum sample (**lower**). Fipronil sulfone is observed at RT = 24.7 min. Only fipronil sulfone is shown because fipronil and fipronil sulfide were not detectable in the serum samples.

The detection limit of the developed method was 5.0, 2.4, and 1.2 ng/L in serum for fipronil, fipronil sulfone, and fipronil sulfide, respectively. These were lower than those reported in the previous studies [7,8] by at least one order of magnitude. As shown in the chromatograms of standard solution (50 ng/L, Figure 1 upper) and a serum sample (Figure 1 lower), there was no apparent interference with fipronil sulfone, indicating that the pretreatment method established in this study could effectively remove interference with fipronil sulfone. The matrix of the deproteinized and solid phase-extracted serum (Control Serum I) was found to decrease the signals of added fipronils by approximately 25%, but this was compensated by the ^13^C-labelled surrogates. Recovery of the added standards (50 ng/L) to the Control Serum I was 97 ± 10, 91 ± 9, and 93 ± 6% for fipronil, fipronil sulfone, and fipronil sulfide, respectively (*n* = 3 each), indicating the satisfactory accuracy (trueness and repeatability) of the method. No fipronils were detected in procedural blanks. The mean and standard deviation of the concentrations of fipronil sulfone in the Control Serum I across 9 baches was 36 ± 3 ng/L, showing satisfactory reproducibility. Overall, the developed method was sensitive and accurate, and it was applied reproducibly throughout the analyses of fipronils in the present serum samples.

### 3.2. Serum Concentrations of Fipronils in Japanese Pregnant Women

Table 2 shows characteristics of the participant pregnant women along with free T4 and TSH levels (n = 131). Since the mean age, pre-pregnancy BMI, primiparous % and passive smoking “yes” % of the participants who donated blood and urine (original participants, n = 231) was 34.1 ± 4.8 years, 20.7 ± 2.4 kg/m^2^, 52%, and 15%, respectively [16], the sub-population of this study was not deviated from the original participant population in terms of these parameters.

**Figure 2:**
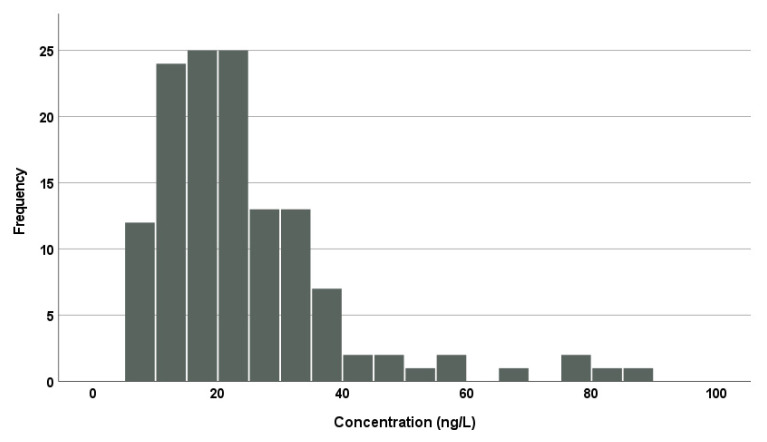
A histogram of fipronil sulfone concentrations in the serum samples of the present study (n = 131).

Table 3 shows average concentrations of fipronil, fipronil sulfone, and fipronil sulfide in the serum of the present participants (n = 131). Fipronil sulfone was detectable in all the participants with the mean (standard deviation) and median (min–max) concentrations being 24 (15) and 21 (6.8–89) ng/L, respectively. Figure 2 shows a histogram of fipronil sulfone concentrations from the present study. The concentrations follow a typical log-normal distribution. In contrast, fipronil and fipronil sulfide were not detectable in any of the participants (<5.0 and <1.2 ng/L, respectively). McMahen et al. [7] analyzed human sera (n = 96) without pesticide exposure and detected fipronil sulfone in 25% of the subjects, though the lower limit of determination was rather high (100 ng/L), but fipronil was hardly detectable. Kim et al. [8] and Shi et al. [9] also reported that fipronil sulfone was detectable in all the subjects from general populations in Korea and China, respectively, while they could hardly detect fipronil and/or fipronil sulfide. Although the detection limits for fipronil and fipronil sulfide were lower by one or two orders of magnitude in our study than those of the previous studies, the two fipronils were still not detectable: a low abundance of the two fipronils in the human serum was confirmed. The distribution pattern of fipronils in the serum indicated a rapid and almost exclusive oxidative conversion from absorbed fipronil to fipronil sulfone and slow elimination of fipronil sulfone, which were observed in rodent studies [10,12,17].

The average serum fipronil sulfone concentration in this study was lower than those in the previous studies: Kim et al. [8] reported median serum concentrations in women who delivered a baby, their husbands, and umbilical cords at the delivery were 753, 1310, and 506 ng/L, respectively, and Shi et al. [9] reported median concentrations of 60–490 ng/L in the adult residents of four cities of China. Although McMahen et al. [7] reported the median concentration to be <100 ng/L, they detected up to 4000 ng/L among non-exposed people, which was far higher than the present maximum (89 ng/L). The reason(s) for the fairly large difference in the average fipronil sulfone concentrations in our study and the previous studies cannot be specified at present. Use of fipronil started in 1996 in Japan, and its shipping amount peaked in 2010, which was followed by continuous decline [18]. In this sense, fipronil exposure in the Japanese general public at the time of blood sampling in this study (2009–2011) was estimated to be greatest. The source of fipronils exposure for the general public is believed to be food and indoor environments [19]; thus, the variation in the abundance of fipronils in these media could be the source of the difference in exposure levels of the populations. Residue levels of fipronils in agricultural products as well as food habits would be different depending on the subject populations. The variation in pet hygiene practice and household pest control would be a significant source of the observed considerable variation in fipronils levels in house dust [3,4,5], which would vary the levels of oral, inhalation, and dermal exposure for people depending on the population. Another reason for the low fipronil sulfone concentrations in this study may be related to the long storage time of the serum samples; in fact, the serum samples analyzed in this study were stored in a freezer at −20 °C for more than 10 years after they had experienced one freeze–thaw cycle. For the present, there is no indication of degradation of the samples during storage though the possibility cannot be excluded. It must be noted that the commercial human serum used for the internal quality control of this study (Control Serum I, lot # 1924_1) had more than 10 freeze–thaw cycles during this study (over three years), but there was no significant change in fipronil sulfone concentrations.

### 3.3. Variation of Serum Fipronil Sulfone Due to Attributes and Dietary Habits of Participants

There was no significant correlation between serum fipronil sulfone levels and age, pre-pregnancy BMI, parity, smoking, and passive smoking.

**Figure 3:**
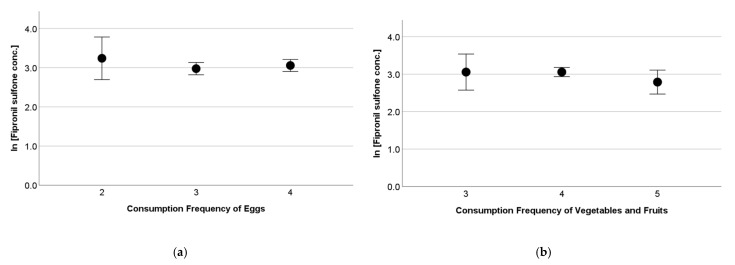
Variation of serum fipronil sulfone concentrations (log-transformed) due to consumption frequency of (**a**) eggs and (**b**) vegetables and fruits of pregnant women. Dot and bar indicate mean and 95% confidence interval, respectively. Horizontal axis denotes 2: less than once a week; 3: 2–3 times a week; 4: every day; 5: every meal. Analysis of variance indicated no significant variation in serum fipronil sulfone concentrations due to consumption frequency of eggs and vegetables and fruits.

There was no significant variation due to the consumption frequency of a food category when tested with an analysis of the variance. Figure 3 shows a variation in serum fipronil sulfone concentrations due to the consumption frequency of eggs and that of vegetables and fruits, as examples. In a Chinese total-diet study, eggs (55.3%), vegetables (30.7%), and meats (5.90%) were found to be the major contributors to daily fipronils intake for Chinese people [20]. Of these, eggs and meats predominantly contained fipronil sulfone, and vegetables contained fipronil [20]. If this is also the case in Japan, the absence of variations due to the consumption frequency of these food in the present study suggests that the contribution of other source(s), e.g., the indoor environment, may be great enough to mask the variation in fipronils exposure due to food habits.

### 3.4. Association Between the Levels of TH and Fipronil Sulfone

Table 4 shows the result of a multiple regression analysis using the fT4 level as a dependent variable and the serum fipronil sulfone level and other potential covariates as independent variables. In this analysis, three participants were excluded because they were diagnosed with thyroid-related diseases (e.g., Hashimoto disease) during pregnancy after the enrollment. The fipronil sulfone concentration was not significant for the dependent variable fT4 level, indicating the fipronils exposure level does not affect the TH status of a pregnant woman. Other independent variables were not significant predictors of the fT4 level except for TSH, which was the only significant variable with a negative partial regression coefficient. This was biologically reasonable. The absence of significant predictors was also the case for the analysis using TSH as a dependent variable (Table 5). The results of the two multiple regression analyses indicated that exposure levels of fipronils did not affect TH levels of pregnant women in Tokyo.

The lack of an association between the serum levels of TH and fipronil sulfone may be attributable to the lower exposure levels of fipronils among the participants in this study. Herin et al. [21] found a negative association between the serum levels of TSH and fipronil sulfone in an occupationally exposed population in France. Serum fipronil sulfone concentrations in the French subjects ranged from 0.37 to 42.45 μg/L, being higher than those in our study (6.8 to 89 ng/L: median 21 ng/L) by one to three orders of magnitude. Kim et al. [8] found a negative association between the levels of T3 and fipronil sulfone (0.159–1.75 ng/mL) in the umbilical cord serum of newborn babies in Korea. Although the direction of the effects seem reversed in the French and Korean studies, and thus the mechanisms of the effects might be different; these studies suggested that there may be some TH disruption in the subjects with higher, at least by one order of magnitude, serum fipronil sulfone levels than those in our study.

As discussed above, the reason(s) for lower fipronil exposure levels in the participants in our study is not specified at present. No aggregated exposure assessment of fipronils has ever been carried out in the world, but according to Chen et al. [19], food and house dust, but not drinking water nor atmosphere, would contribute to the daily intake of fipronils for the general public. It is supposed that levels of fipronils in foods or in the residential environments of our study participants might be lower than those in other previously reported populations; however, there is no data in Japan as to the levels of fipronils in these media. Since fipronil has not only TH toxicity but also hepatic, renal, and neuronal toxicities, and carcinogenicity, an aggregated exposure assessment would be necessary to fully characterize the exposure for the general public and, consequently, to quantitatively assess its health risk.

### 3.5. Limitations of This Study

Limitations of this study include a moderate number of subjects involved (n = 131), though the number was more than those in any other previous studies. In general, increasing the number of subjects would make detectable a small health effect, if present. A lack of information on environmental exposure to fipronils in Japan may be another limitation because it made it difficult to discuss the serum levels in relation to exposure.

## 4. Conclusions

Serum levels of fipronil and its metabolites were determined in Japanese pregnant women with a sensitive and accurate analytical method developed in our laboratory. Fipronil sulfone was the only one of the fipronils detected in all the participants (median 21 ng/L, n = 131), but fipronil and fipronil sulfide were not detected at all, indicating the rapid and exclusive conversion of fipronil to fipronil sulfone in humans. No attributes or dietary habits investigated in this study were found to be the source(s) of inter-individual variations in serum fipronil sulfone levels. An aggregated exposure assessment is warranted in Japan to make clear the sources and magnitude of exposure to fipronils. Multiple regression analyses of serum free T4 or TSH levels as dependent variables did not find the serum fipronil sulfone level as a significant independent variable, indicating fipronil or fipronil sulfone exposure at these exposure levels do not disrupt circulating TH levels. The present result would be valuable for assessing a dose–effect relationship of fipronils in humans on population levels.

## Figures and Tables

**Table 1:** Settings of mass spectrometers.

LC-MS/MS Parameter						
Instrument		Triple Quad 5500+ QTRAP Ready (SCIEX)				
Ionization mode		ESI, negative mode				
Ionization splay voltage (V)		−4500				
Ion source temperature (°C)		300				
Monitored ion and MS voltage setting						
	Ion (*m*/*z*)			Voltage setting (V)		
	Precursor	Product	Qualifier	Declustering potential	Collision energy	Collision cell exit
Fipronil	435.7	250.9	330.5	−105	−40	−25
Fipronil sulfone	451.9	282.9	415.8	−105	−40	−13
Fipronil sulfide	419.9	262.9	383.8	−90	−40	−19
^13^C-fipronil	441.9	336.8	252.8	−100	−24	−33
^13^C-fipronil sulfone	457.9	421.8	288.8	−110	−24	−33
^13^C-fipronil sulfide	425.9	389.8	265.2	−95	−20	−19
Chromatographic conditions						
Column		InertSustain C18, 5 µm, 150 × 2.1 mm ID (GL Science)				
Column temperature (°C)		40				
Mobile phase gradient A: Methanol B: 0.05 mM Ammonium fluoride		0–1 min A 20% B 80% 1–25 min A 20–90% B 80–10% 25–30 min A 90% B 10% 30–35 min A 20% B 80%				
Mobile phase flow rate (mL/min)		0.2				
Injection (µL)		8.0				

**Table 2:** Biological attributes and thyroid hormone status of the participants (n = 131).

	Unit	N	Mean (Standard Deviation)	Min–Max
Age	Yrs	128	34.1 (4.8)	22–48
Pre-pregnancy BMI	kg/m^2^	129	21.1(2.7)	17.1–35.0
Urinary iodine	μg/g cre	121	390 (2.7) *	47–6159
fT4	ng/dL	131	1.24 (1.21) *	0.83–3.41
TSH	μIU/mL	116	0.651 (4.56) *^#^	<0.005–27.4
Parity		Primiparous 65 (50%) Multiparous 64 (50%)		
Smoking by the time of pregnancy diagnosis		Yes 11 (8.8%) No 114 (91.2%)		
Current passive smoking		Yes 25 (22%) No 89 (78%)		

* Geometric mean (geometric standard deviation). ^#^ Mean was calculated by substituting undetectable concentration (<0.005) with half of detection limit value (0.0025).

**Table 3:** Serum concentrations of fipronils * (n = 131).

	Detection Frequency (%)	Mean (SD)	Geometric Mean (SD)	Median	Min–Max
Fipronil	0	<0.005	<0.005	<0.005	
Fipronil sulfone	100	24 (15)	21 (1.7)	21	6.8–89
Fipronil sulfide	0	<0.001	<0.001	<0.001	

* Unit of the figures in the table is ng/L unless otherwise indicated.

**Table 4:** Result of multiple regression analysis (dependent variable: serum free T4).

Dependent Variable: Serum Free T4 *			
Independent Variable	β (95% CI)	Adjusted β	*p*
Fipronil sulfone *	0.010 (−0.033–0.052)	0.039	0.65
Age	−0.001 (−0.005–0.004)	−0.020	0.81
BMI	0.001 (−0.009–0.010)	0.014	0.87
Parity	−0.014 (−0.062–0.034)	−0.049	0.58
Urinary iodine *	0.005 (−0.019–0.030)	0.037	0.66
Smoking until pregnancy	−0.001 (−0.090–0.089)	−0.002	0.99
Passive smoking	−0.015 (−0.079–0.049)	−0.044	0.64
TSH *	−0.089 (−0.107–0.071)	−0.716	<0.001
Constant	0.133 (−0.209–0.475)		0.44
Model F = 8.282 *p* < 0.001 Adjusted R^2^: 0.380			

* Log-transformed.

**Table 5:** Result of multiple regression analysis (dependent variable: serum TSH).

Dependent Variable: Serum TSH *			
Independent Variable	β (95% CI)	Adjusted β	*p*
Fipronil sulfone *	0.088 (−0.290–0.467)	0.039	0.64
Age	0.014 (−0.028–0.057)	0.055	0.50
BMI	−0.063 (−0.147–0.021)	−0.124	0.14
Urinary iodine *	0.047 (−0.172–0.266)	0.036	0.67
Parity	−0.056 (−0.487–0.375)	−0.022	0.80
Smoking until pregnancy	−0.115 (−0.917–0.688)	−0.025	0.78
Passive smoking	0.226 (−0.344–0.795)	0.072	0.43
Serum free T4 *	−5.688 (−7.171–4.214)	−0.623	<0.001
Constant	1.184 (−1.879–4.247)		0.44
Model F = 9.033 *p* < 0.001 Adjusted R^2^: 0.495			

* Log-transformed.

## Data Availability

Data obtained during this study are not publicly available because of ethical reasons, but they are available upon reasonable request to the corresponding author.
